# Latent profile analysis of trait impulsivity facets and associations with resilience, problematic alcohol use, and quality of life

**DOI:** 10.1111/acer.70158

**Published:** 2025-09-09

**Authors:** Heidi H. Meyer, Matthew F. Thompson, Tommy Gunawan, Melanie L. Schwandt, Vijay A. Ramchandani, Nancy Diazgranados, Jeremy W. Luk

**Affiliations:** ^1^ Office of the Clinical Director National Institute on Alcohol Abuse and Alcoholism Bethesda Maryland USA; ^2^ Department of Psychiatry & Human Behavior The Warren Alpert Medical School of Brown University Providence Rhode Island USA; ^3^ Human Psychopharmacology Laboratory National Institute on Alcohol Abuse and Alcoholism Bethesda Maryland USA

**Keywords:** impulsivity, negative urgency, positive urgency, quality of life, resilience

## Abstract

**Background:**

Impulsivity is a multidimensional construct that is associated with problematic alcohol use and alcohol use disorder (AUD). Modeling within‐person clustering of impulsivity facets has the potential to aid clinical case conceptualization, and examining associations with resilience and well‐being outcomes can inform strength‐based intervention approaches. In this study, we utilized latent profile analysis (LPA) to capture the clustering of trait impulsivity facets and tested resilience as a mediational pathway linking impulsivity latent profiles to problematic alcohol use and quality of life domains.

**Methods:**

A total of 401 adults (59.9% male and 71.6% with past‐year AUD) who completed self‐reported measures of trait impulsivity (Barratt Impulsiveness Scale and UPPS‐P Impulsive Behavior Scale), resilience, and alcohol‐related outcomes were included in this study. Statistical analyses included LPA, linear regression, and path analysis.

**Results:**

LPA identified three profiles that varied by overall impulsivity as well as specific levels of negative and positive urgency: Profile 1—Low Impulsivity/Urgency (36.4%), Profile 2—Medium Impulsivity (45.6%), and Profile 3—High Impulsivity/Urgency (18.0%). The percentages of past‐year AUD were 37.7% in Profile 1, 87.4% in Profile 2, and 100% in Profile 3. Latent profiles with higher impulsivity had lower resilience, higher problematic alcohol use, and lower quality of life. Low resilience was a significant mediator of associations between Medium/High Impulsivity profiles and all clinical outcomes, including problematic alcohol use and four quality of life domains.

**Conclusions:**

In this person‐centered analysis, individuals who scored high on negative urgency also had elevated scores on positive urgency and several other impulsivity facets. Within‐person clustering of impulsivity facets was associated with differential risk for AUD, and latent profile differences in problematic alcohol use and quality of life outcomes were mediated by low resilience. Findings highlight resilience as a potential treatment target that warrants further evaluation in clinical research.

## Graphical abstract

Using latent profile analysis, we found substantial within‐person clustering of eight trait impulsivity facets, including negative and positive urgency. With 100% precision, individuals who were classified into the latent profile with high impulsivity/urgency (18% of the sample) all met criteria for past‐year AUD. In path analysis, resilience partially mediated the associations of medium/high impulsivity profiles with problematic alcohol use and quality of life outcomes. Strength‐based AUD intervention approaches that promote resilience hold promise and should be further investigated.

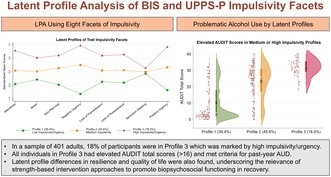

## INTRODUCTION

Alcohol is one of the most used substances in the United States, with approximately 85% of adults drinking at some point in their life (Substance Abuse and Mental Health Services Administration, 2022). It is estimated that alcohol use disorder (AUD) affects approximately 29 million individuals in the United States (Substance Abuse and Mental Health Services Administration, 2023). AUD is associated with adverse physical health conditions, psychiatric comorbidities, impaired quality of life, and the burden of disease measured in terms of years of life lost due to mortality or disability is substantial (Rehm et al., 2014; Schuckit, 2009). AUD also has significant economic costs for society related to healthcare, lost productivity, and crime (Sacks et al., 2015). To prevent alcohol misuse and the adverse impacts associated with AUD, it is important to identify risk and protective pathways leading to problematic alcohol use.

Trait impulsivity, referring to the dispositional tendency to act rashly without considering potential consequences, is a well‐established risk factor for AUD (Dick et al., 2010; Stamates & Lau‐Barraco, 2017) and related conditions (Curry et al., 2018; Luk et al., 2017; Thompson et al., 2025). Measures of trait impulsivity such as the Barratt Impulsiveness Scale (BIS) (Patton et al., 1995) and the UPPS‐P Impulsive Behavior Scale (UPPS‐P) (Whiteside & Lynam, 2001) recognize impulsivity as a multidimensional construct. A meta‐analysis of 96 studies focusing on the UPPS‐P and alcohol outcomes found that lack of perseverance was most strongly associated with drinking quantity, whereas negative and positive urgency were most strongly associated with drinking problems (Coskunpinar et al., 2013). However, as impulsivity facets are often moderately to highly correlated with one another (Adams et al., 2012; Curcio & George, 2011; Cyders et al., 2009), estimating the unique association of one impulsivity facet with problematic alcohol use while controlling for all the other facets is suboptimal because the inferences drawn are focused on the relationships among variables rather than on the clinical presentation of individuals. In contrast, modeling within‐person clustering of impulsivity facets has the potential to aid clinical case conceptualization (e.g., elevated scores on specific and/or multiple impulsivity facets may indicate heightened risk for problematic alcohol use and AUD), and examining associations with resilience and well‐being outcomes can inform strength‐based intervention approaches.

Latent profile analysis (LPA) is a person‐centered approach that can be utilized to identify subgroups of individuals who share characteristics (e.g., personality traits) that are not directly observed (Nylund‐Gibson & Choi, 2018). Though not examined in the current study, it is of value to note that LPA has been widely applied to study the clustering of the Big Five personality traits (openness, conscientiousness, extraversion, agreeableness, and neuroticism) to identify latent profiles and their associations with psychological functioning and well‐being outcomes (Merz & Roesch, 2011; Ratchford et al., 2022; Specht et al., 2014). A systematic review of 34 empirical studies with 36 independent samples showed that three‐profile or four‐profile solutions were most common for the Big Five personality traits and that the neuroticism facet was most differentiated across latent profiles (Yin et al., 2021). These studies illustrate the feasibility of using LPA to identify distinct and interpretable latent profiles within a sample using as few as five personality trait indicators.

Recent research has begun utilizing LPA to capture impulsivity risk profiles that account for within‐person variation in different facets of impulsivity. For example, in a sample of 360 college students, Stamates et al. (2021) found three UPPS‐P latent profiles that differed based on levels of impulsivity, supporting the idea that impulsivity facets tend to cluster together to confer risk for negative drinking motives and alcohol consequences. In another sample of 599 college students, Moussa Rogers et al. (2021) identified five UPPS‐P latent profiles, with one characterized by elevations in lack of premeditation and perseverance, and two other profiles characterized by elevations in sensation seeking and urgency facets. These studies demonstrate the utility of LPA in identifying distinct impulsivity profiles and their associations with alcohol and other substance use; however, findings are limited by the focus on college student samples and just the five UPPS‐P facets. To address these limitations, the current study utilized LPA to capture trait impulsivity latent profiles in a heterogeneous sample of adults across the spectrum of AUD and with a broader range of impulsivity indicators drawn from both the BIS and the UPPS‐P. The inclusion of eight facets of impulsivity as indicators in the LPA would distinguish this study as a novel investigation rather than a direct replication of prior research. In addition, having more impulsivity indicators in the LPA may be beneficial from a modeling perspective given the additional data provided to facilitate model estimation and identification.

Identifying impulsivity profiles can facilitate the examination of their associations with problematic alcohol use and quality of life. Prior research has documented risk pathways that link trait impulsivity to alcohol‐related outcomes through factors such as peer substance use (Barnow et al., 2004), alcohol expectancy (Gullo et al., 2010), and impaired control over drinking (Vaughan et al., 2019; Wardell et al., 2016). Findings from these studies may inform clinical strategies that encourage individuals with elevated impulsivity levels to avoid high‐risk social situations and challenge maladaptive thoughts associated with drinking behaviors (Liese & Beck, 2022). In comparison, limited research has tested resilience as a potential pathway linking trait impulsivity to alcohol‐related outcomes. This is a significant research gap considering current proposals to utilize positive psychology interventions in the prevention and intervention of alcohol misuse given their easily accessible and scalable nature (Carlon et al., 2025; Chakhssi et al., 2018; Krentzman, 2013).

Resilience, referring to the ability to positively adapt to adversity, is a strength‐based factor that can be targeted and improved through resilience training or intervention (Joyce et al., 2018; Liu et al., 2020; Meyer & Stutts, 2024). A meta‐analytic review of 30 studies found that positive personality traits from the Big Five (i.e., agreeableness, conscientiousness, extraversion, and openness) were associated with higher resilience (*r*_s_ ranged from 0.31 to 0.42), whereas neuroticism was inversely associated with resilience (*r* = −0.46) (Oshio et al., 2018). However, parallel research on associations between resilience and impulsivity facets remains scant. Given the protective role of resilience against risky alcohol consumption (Cusack et al., 2023) and the implications of resilience on stress management and addiction treatment (Schwandt et al., 2024), further research is warranted to assess potential indirect associations between impulsivity and alcohol‐related outcomes through low resilience. In addition, consistent with recent research that has highlighted biopsychosocial functioning as an integral part of recovery (Hagman et al., 2022; Luk et al., 2022, 2024), multidimensional quality of life domains were also examined as secondary outcomes.

In this study, we utilized a person‐centered approach to capture distinct profiles of impulsivity facets and examined latent profile differences in resilience, problematic alcohol use, and quality of life domains. We further tested resilience as a potential mediator linking the impulsivity latent profiles to problematic alcohol use and quality of life. Referencing prior latent profile analyses of the UPPS‐P, we hypothesized that at least three latent profiles would emerge, showing variation in both overall level of impulsivity and patterns of specific facets of impulsivity, and that profiles with higher impulsivity would report lower resilience, more problematic alcohol use, and lower quality of life. We also hypothesized that resilience would be a significant mediator of the associations between latent profiles and alcohol‐related outcomes.

## METHOD

### Participants

The study sample included a total of 401 adults who completed the National Institute on Alcohol Abuse and Alcoholism (NIAAA) Addictions Neuroclinical Assessment (ANA) protocol. To be eligible for the ANA protocol, participants must be at least 18 years old and must also be enrolled in the NIAAA Natural History Protocol, which required participants to be not under legal confinement and not pregnant or breastfeeding. The goals of the NIAAA Natural History Protocol were to gather data on a wide range of behavioral traits and clinical characteristics among individuals with or without alcohol use problems and to determine participants' potential eligibility for other research protocols conducted at the NIH Clinical Center. As part of the Natural History Protocol, participants completed a standardized battery of assessments and questionnaires, including self‐report measures of impulsivity, problematic alcohol use, and quality of life. Participants were also evaluated for AUD by trained clinicians using the Structured Clinical Interview for DSM‐5 (First, 2015). In the current sample, 71.6% of the study participants (*n* = 287) met criteria for past‐year AUD, and 28.4% (*n* = 114) did not meet criteria. The high proportion of study participants with AUD may be related to the availability of an inpatient AUD rehabilitation treatment program at the NIH Clinical Center. Data on resilience were drawn from the ANA protocol, whereas all other measures were drawn from the Natural History Protocol. The study protocols were approved by the NIH Intramural Institutional Review Board and have been registered on clinicaltrials.gov (NCT02231840; NCT04946851). Informed consent was obtained from all participants before they completed the clinical assessments at the NIH Clinical Center.

### Measures

Measures of demographic characteristics, trait impulsivity, resilience, and clinical outcomes are described below. For internal consistency reliability, Cronbach's alphas and McDonald's omegas for the impulsivity facets, resilience, problematic alcohol use, and quality of life domains are presented in Table 1.

**TABLE 1:** Cronbach's alphas and McDonald's omegas for study measures.

	Cronbach's alphas	McDonald's omegas
Barratt Impulsiveness Scale		
Attentional	0.82	0.82
Motor	0.64	0.70
Non‐planning	0.80	0.81
UPPS‐P Impulsive Behavior Scale		
Negative urgency	0.93	0.93
Lack of premeditation	0.87	0.88
Lack of perseverance	0.85	0.86
Sensation seeking	0.86	0.86
Positive urgency	0.96	0.96
Connor‐Davidson Resilience Scale	0.92	0.93
Alcohol Use Disorders Identification Test	0.94	0.94
World Health Organization Quality of Life‐BREF		
Physical	0.82	0.82
Psychological	0.89	0.89
Social	0.75	0.77
Environment	0.85	0.86

### Demographic characteristics

Participants completed self‐report measures of age, sex, race, marital status, years of education, and annual household income.

### Trait impulsivity

Eight facets of trait impulsivity, drawn from the Barratt Impulsiveness Scale (BIS‐11) and the UPPS‐P Impulsive Behavior Scale (UPPS‐P), were used as indicators of impulsivity in the LPA. The BIS‐11 (Barratt, 1994) uses 30 self‐report items to assess impulsivity and has three subscales capturing attentional, motor, and nonplanning impulsiveness. Participants responded to all items on a 4‐point Likert scale from 1 (Rarely/Never) to 4 (Almost Always/Always). The UPPS‐P (Cyders et al., 2007) also measures multidimensional impulsivity, but through 59 self‐report items, rated from 1 (Agree Strongly) to 4 (Disagree Strongly). This scale is an updated version of the original UPPS, which had only four facets (Whiteside & Lynam, 2001). The UPPS‐P is comprised of five facets of impulsivity: negative urgency, lack of premeditation, lack of perseverance, sensation seeking, and positive urgency. Across all facets of impulsivity, higher scores indicate higher impulsivity.

### Resilience

The Connor‐Davidson Resilience Scale (CDRS) is a validated measure comprised of 25 self‐report items (Connor & Davidson, 2003). Items were rated on a 5‐point Likert scale from 0 (Not True at All) to 4 (True Nearly All of the Time). Total scores, computed as the sum of the 25 item scores, range from 0 to 100, with higher scores reflecting higher resilience.

### Problematic alcohol use

The Alcohol Use Disorders Identification Test (AUDIT) was used to assess problematic alcohol use within the past year. This screening tool was developed by the World Health Organization to assess alcohol consumption, alcohol‐related harms, and alcohol‐related problems (Saunders et al., 1993). Its 10 items are summed for a total score, with a score of 8–15 considered hazardous alcohol use (may indicate a need for brief counseling), whereas a score of 16 or higher is indicative of high‐risk drinking (may indicate a need for more structured intervention and potential referral to specialized treatment) (Babor et al., 2001).

### Quality of life

The World Health Organization Quality of Life‐BREF scale (WHOQOL‐BREF) was used to measure quality of life within the past 2 weeks. This widely used scale provides scores for four domains: physical, psychological, social, and environmental quality of life (World Health Organization, 1998). Items for each domain were summed and then standardized from 0 to 100, with higher scores representing higher quality of life.

### Statistical analyses

Data analysis consisted of three steps. First, LPA was applied to eight impulsivity facets (three from the BIS and five from the UPPS‐P) to determine impulsivity profiles within the sample. Specifically, all eight impulsivity facets were first standardized (with a mean of zero and a standard deviation of one) and a series of one‐profile to five‐profile models was run and compared using standard model fit statistics including AIC (Akaike Information Criterion), BIC (Bayesian Information Criterion), entropy, and likelihood ratio tests (Vuong–Lo–Mendell–Rubin and Lo–Mendell–Rubin likelihood ratio tests). The selection of the best‐fitting profile was based on model fit statistics, model parsimony, and interpretability of the latent profiles (Nylund et al., 2007; Nylund‐Gibson & Choi, 2018). Second, the associations of the identified impulsivity profiles with resilience, problematic alcohol use, and quality of life domains were examined using the three‐step approach, which accounts for the posterior probabilities in profile classification (Asparouhov & Muthén, 2014), as well as linear regression analyses using the extracted impulsivity profiles. Third, a path analysis model was specified to test resilience as a potential mediator of the associations between impulsivity profiles and clinical outcomes. Study covariates were selected a priori based on theoretical considerations while referencing a person‐centered analysis conducted during the COVID‐19 pandemic (Luk et al., 2023). The final list of covariates, which included age, sex, race, marital status, years of education, and annual household income, were further supported on a statistical basis as each of these demographic characteristics was significantly associated with one or more study outcomes. In the adjusted analysis, all study covariates were entered into the multivariable model in a single step. As a comparison with the LPA approach, we conducted a supplemental analysis using the traditional variable‐centered regression approach to illustrate similarities and differences between the two approaches. Statistical analyses were conducted using Stata 19.0 and Mplus 8.4.

## RESULTS

### Sample characteristics

The study sample had a mean age of 42.5 years (SD = 13.0). Demographic characteristics for the overall sample and by latent profile are shown in Table 2. The study sample was 59.9% male, 51.9% White, and 61.9% single. Most participants reported 13 to 16 years of education (53.6%) and an annual household income of $20,000 to $74,999 (41.2%). In addition, the mean AUDIT score for the sample was 20.0 (SD = 12.7), and the majority of participants (71.6%) had a current diagnosis of AUD in the past year.

**TABLE 2:** Demographic characteristics in the overall sample and by latent profiles.

	Overall sample (*n* = 401)		Latent Profile 1: low impulsivity/urgency (*n* = 146)		Latent Profile 2: medium impulsivity (*n* = 183)		Latent Profile 3: high impulsivity/urgency (*n* = 72)	
	Mean	SD	Mean	SD	Mean	SD	Mean	SD
Age (years)	42.5	13.0	42.1	14.1	43.2	12.8	41.7	11.5
	**Frequency**	**Percent**	**Frequency**	**Percent**	**Frequency**	**Percent**	**Frequency**	**Percent**
Sex								
Female	161	40.1%	60	41.1%	67	36.6%	34	47.2%
Male	240	59.9%	86	58.9%	116	63.4%	38	52.8%
Race								
White	208	51.9%	70	48.0%	90	49.2%	48	66.7%
Black	139	34.7%	58	39.7%	66	36.1%	15	20.8%
Other	54	13.5%	18	12.3%	27	14.8%	9	12.5%
Marital status								
Single	248	61.9%	92	63.0%	118	64.5%	38	52.8%
Married	73	18.2%	36	24.7%	24	13.1%	13	18.1%
Other	80	20.0%	18	12.3%	41	22.4%	21	29.2%
Years of education								
Fewer than 13 years	122	30.4%	20	13.7%	74	40.4%	28	38.9%
13–16 years	215	53.6%	87	59.6%	90	49.2%	38	52.8%
17 or more years	64	16.0%	39	26.7%	19	10.4%	6	8.3%
Annual household income								
Below $20,000	117	29.2%	24	16.4%	61	33.3%	32	44.4%
$20,000–$74,999	165	41.1%	59	40.4%	81	44.3%	25	34.7%
$75,000 or higher	119	29.7%	63	43.2%	41	22.4%	15	20.8%
AUD in the past year								
No AUD	114	28.4%	91	62.3%	23	12.6%	0	0.0%
AUD	287	71.6%	55	37.7%	160	87.4%	72	100.0%

*Note*: The high proportion of study participants with AUD may be related to the availability of an inpatient AUD rehabilitation treatment program at the NIH Clinical Center.

### Latent profile analysis

**FIGURE 1:**
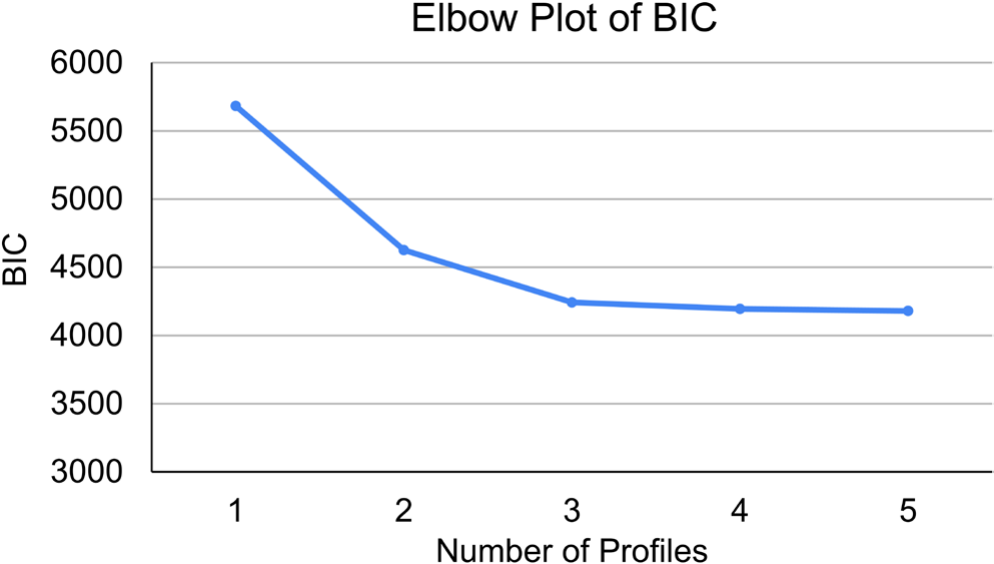
Elbow plot. Elbow plot of Bayesian information criterion (BIC) shows that increasing the number of profiles yielded substantial decreases in BIC up to three profiles, and the reductions in BIC were much smaller when modeling four and five profiles.

Table 3 shows the model fit statistics by the number of latent profiles. Both AIC and BIC exhibited substantial decreases from 1 to 2 and 2 to 3 profiles, whereas decreases in AIC and BIC from 3 to 4 profiles were smaller (see Figure 1 for an elbow plot of the BIC values by number of profiles). The three‐profile solution yielded the highest entropy (0.896), and the Vuong–Lo–Mendell–Rubin and Lo–Mendell–Rubin likelihood ratio tests indicated that a four‐profile solution did not substantially improve model fit. Taken together, the model fit statistics, model parsimony, and interpretability of the latent profiles indicated that a three‐profile solution was optimal within the current sample.

**TABLE 3:** Model fit statistics by number of latent profiles.

Criteria	Number of latent profiles				
	1	2	3	4	5
Log likelihood	−2793.254	−2237.972	−2019.296	−1968.433	−1934.052
Akaike information criterion (AIC)	5618.508	4525.944	4106.592	4022.866	3972.104
Bayesian information criterion (BIC)	5682.411	4625.793	4242.387	4194.606	4179.790
Sample‐size‐adjusted BIC	5631.642	4546.466	4134.502	4058.163	4014.790
Entropy	N/A	0.862	0.896	0.834	0.826
Vuong–Lo–Mendell–Rubin likelihood ratio test					
*p* Value	N/A	0.0002	<0.0001	0.1351	0.3922
Lo–Mendell–Rubin likelihood ratio test					
*p* Value	N/A	0.0002	<0.0001	0.1396	0.3968
Profile proportions		54.6%	36.4%	28.7%	24.9%
		45.4%	45.6%	13.2%	15.7%
			18.0%	27.2%	15.5%
				30.9%	32.7%
					11.2%

*Note*: The three‐profile solution was selected as the best‐fitting model with the lowest BIC and well‐differentiated profiles that are highly interpretable. The Vuong–Lo–Mendell–Rubin and the Lo–Mendell–Rubin likelihood ratio tests provided further support for the three‐profile model as it was found to be superior to the two‐profile model, and the four‐profile model was not superior to the three‐profile model.

**FIGURE 2:**
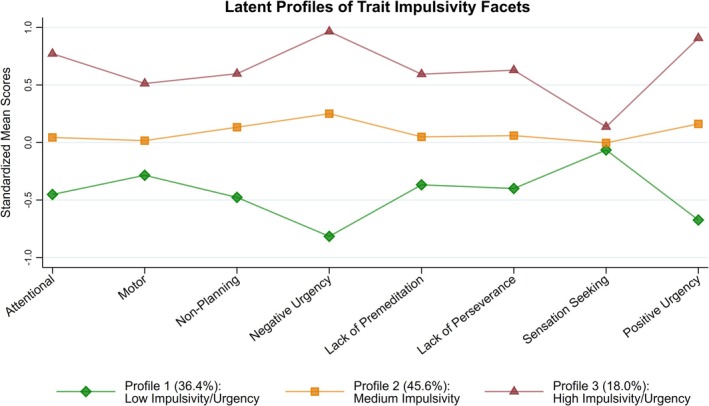
Latent profile plot. Standardized means of impulsivity facets for each latent profile are presented.

Figure 2 shows the standardized means for each facet of impulsivity across the three latent profiles. Profile 1 (*n* = 146; 36.4%) had the lowest standardized means across all indicators, and especially for negative and positive urgency, with scores being more than half a standard deviation below the mean. Therefore, Profile 1 was labeled as Low Impulsivity/Urgency. The standardized means of all indicators for Profile 2 (*n* = 183; 45.6%) were around zero, and Profile 2 was labeled as Medium Impulsivity. Profile 3 (*n* = 72; 18.0%) had the highest standardized means across all indicators, and particularly so for negative and positive urgency, with scores being close to one standard deviation above the mean. Therefore, Profile 3 was labeled as High Impulsivity/Urgency. Overall, the latent profiles were most differentiated on negative and positive urgency, and least differentiated on sensation seeking. The percentages of past‐year AUD were 37.7% in Profile 1, 87.4% in Profile 2, and 100% in Profile 3. In terms of AUDIT scores, 85.2% of those with Medium Impulsivity (Profile 2) had an AUDIT score of 8 or higher, and 100% of those with High Impulsivity (Profile 3) had an AUDIT score of 16 or higher.

### Latent profile differences in resilience, problematic alcohol use, and quality of life

**FIGURE 3:**
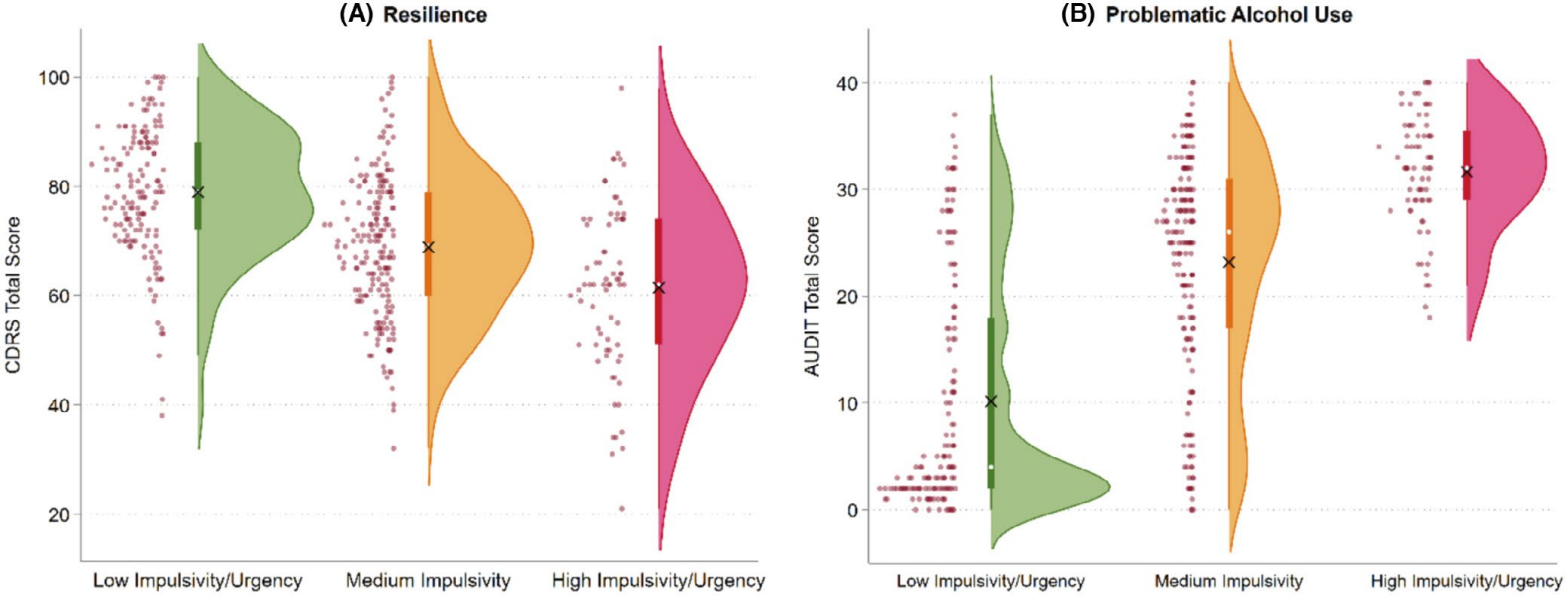
Raincloud plots for resilience and problematic alcohol use. Raincloud plots are utilized to show the distribution of the data. For each distribution, the white dot represents the median, and the × symbol represents the mean.

Consistent with a high entropy value of 0.896, results from the three‐step approach (see Table 4) were highly similar to those using the extracted latent profiles. Latent profile differences in resilience and problematic alcohol use are visually displayed in Figure 3. Compared with Profile 1, CDRS scores (Panel A) were lower in Profile 2 (*b* = −10.09, 95% confidence intervals [CI] = −12.92, −7.25) and Profile 3 (*b* = −17.45, 95% CI = −21.13, −13.76). For problematic alcohol use (Panel B), AUDIT scores were higher in Profile 2 (*b* = 13.03, 95% CI = 10.87, 15.19) and Profile 3 (*b* = 21.51, 95% CI = 18.70, 24.31) than in Profile 1. These findings indicated that clustering of impulsivity facets as captured by the latent profiles was inversely associated with resilience and was strongly associated with problematic alcohol use.

**TABLE 4:** Results from the three‐step approach to estimate latent profile differences in resilience and clinical outcomes.

	Mean	SE	Pairwise comparisons	Chi‐squared	*p*
Resilience					
Profile 1	79.85	1.11	Profile 2 vs. 1	49.05	<0.001
Profile 2	68.46	1.03	Profile 3 vs. 1	75.62	<0.001
Profile 3	61.26	1.84	Profile 3 vs. 2	11.12	0.001
Problematic alcohol use					
Profile 1	8.95	1.34	Profile 2 vs. 1	62.70	<0.001
Profile 2	23.60	0.91	Profile 3 vs. 1	234.29	<0.001
Profile 3	31.87	0.66	Profile 3 vs. 2	52.79	<0.001
Physical quality of life					
Profile 1	86.10	1.55	Profile 2 vs. 1	61.97	<0.001
Profile 2	68.61	1.29	Profile 3 vs. 1	144.05	<0.001
Profile 3	57.05	1.88	Profile 3 vs. 2	23.76	<0.001
Psychological quality of life					
Profile 1	79.88	1.11	Profile 2 vs. 1	136.47	<0.001
Profile 2	58.08	1.47	Profile 3 vs. 1	181.67	<0.001
Profile 3	40.16	2.74	Profile 3 vs. 2	29.27	<0.001
Social quality of life					
Profile 1	73.50	1.81	Profile 2 vs. 1	42.13	<0.001
Profile 2	56.24	1.75	Profile 3 vs. 1	74.49	<0.001
Profile 3	44.36	2.86	Profile 3 vs. 2	11.80	0.001
Environment quality of life					
Profile 1	79.03	1.28	Profile 2 vs. 1	73.67	<0.001
Profile 2	62.20	1.41	Profile 3 vs. 1	75.72	<0.001
Profile 3	55.30	2.41	Profile 3 vs. 2	5.47	0.019

*Note*: The three‐step approach in Mplus was used to account for posterior probabilities of profile membership when estimating associations between latent profiles and variables of interest.

**FIGURE 4:**
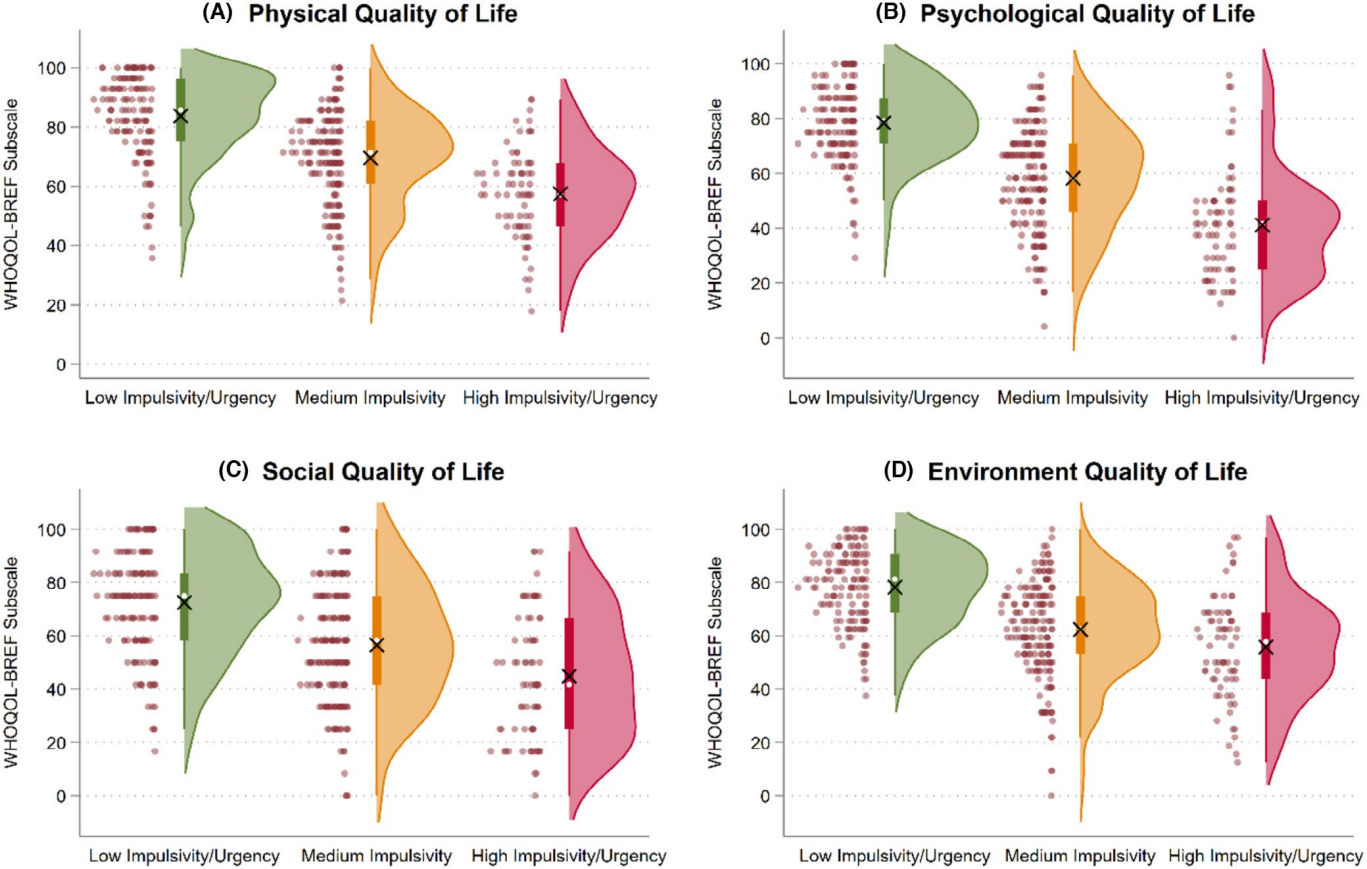
Raincloud plots for four domains of quality of life. Raincloud plots are utilized to show the distribution of the data. For each distribution, the white dot represents the median and the × symbol represents the mean.

Latent profile differences in quality of life are consistent across the four domains and are visually displayed in Figure 4. For physical quality of life (Panel A), subscale scores were lower in Profile 2 (*b* = −14.22, 95% CI = −17.55, −10.88) and Profile 3 (*b* = −26.29, 95% CI = −30.62, −21.96) than in Profile 1. For psychological quality of life (Panel B), subscale scores were lower in Profile 2 (*b* = −20.24, 95% CI = −24.01, −16.46) and Profile 3 (*b* = −37.34, 95% CI = −42.24, −32.44) than in Profile 1. For social quality of life (Panel C), subscale scores were also lower in Profile 2 (*b* = −15.98, 95% CI = −20.70, −11.25) and Profile 3 (*b* = −27.70, 95% CI = −33.83, −21.56) than in Profile 1. Finally, for environment quality of life (Panel D), subscale scores were lower in Profile 2 (*b* = −15.83, 95% CI = −19.51, −12.15) and Profile 3 (*b* = −22.48, 95% CI = −27.26, −17.70) than in Profile 1. These findings showed that clustering of impulsivity facets as captured by the latent profiles was inversely associated with all four quality of life domains.

### Path analysis and the mediating role of resilience

Resilience was tested as a potential mediator of the associations between Medium/High Impulsivity Profiles and clinical outcomes, including problematic alcohol use and the four quality of life domains. Table 5 shows the indirect, direct, and overall associations of impulsivity latent profiles with the clinical outcomes. All indirect associations were statistically significant and supported the role of resilience as a mediator, though none attenuated the direct associations between Medium/High Impulsivity Profiles and clinical outcomes. The proportion of the overall effects mediated by resilience ranged from 8.7% to 9.1% for problematic alcohol use and ranged from 18.1% to 27.3% for quality of life domains.

**TABLE 5:** Results from the path analysis.

Mediational pathways	Effects	*b* (95% confidence intervals)	*p*	Proportion mediated
Profile 2 → Resilience → Problematic alcohol use	Indirect	0.039 (0.014, 0.071)	0.007	9.1%
	Direct	0.391 (0.285, 0.488)	<0.001	
	Total	0.430 (0.329, 0.522)	<0.001	
Profile 3 → Resilience → Problematic alcohol use	Indirect	0.050 (0.017, 0.093)	0.009	8.7%
	Direct	0.523 (0.432, 0.618)	<0.001	
	Total	0.573 (0.489, 0.660)	<0.001	
Profile 2 → Resilience → Physical quality of life	Indirect	−0.071 (−0.116, −0.041)	<0.001	21.5%
	Direct	−0.259 (−0.353, −0.163)	<0.001	
	Total	−0.330 (−0.422, −0.234)	<0.001	
Profile 3 → Resilience → Physical quality of life	Indirect	−0.091 (−0.141, −0.054)	<0.001	18.1%
	Direct	−0.411 (−0.509, −0.309)	<0.001	
	Total	−0.503 (−0.603, −0.407)	<0.001	
Profile 2 → Resilience → Psychological quality of life	Indirect	−0.118 (−0.166, −0.079)	<0.001	26.3%
	Direct	−0.330 (−0.403, −0.254)	<0.001	
	Total	−0.448 (−0.524, −0.368)	<0.001	
Profile 3 → Resilience → Psychological quality of life	Indirect	−0.152 (−0.205, −0.108)	<0.001	24.8%
	Direct	−0.460 (−0.552, −0.358)	<0.001	
	Total	−0.612 (−0.695, −0.514)	<0.001	
Profile 2 → Resilience → Social quality of life	Indirect	−0.082 (−0.126, −0.050)	<0.001	27.3%
	Direct	−0.219 (−0.320, −0.123)	<0.001	
	Total	−0.300 (−0.393, −0.201)	<0.001	
Profile 3 → Resilience → Social quality of life	Indirect	−0.105 (−0.163, −0.064)	<0.001	24.4%
	Direct	−0.325 (−0.435, −0.203)	<0.001	
	Total	−0.431 (−0.531, −0.320)	<0.001	
Profile 2 → Resilience → Environment quality of life	Indirect	−0.071 (−0.113, −0.040)	<0.001	22.5%
	Direct	−0.245 (−0.346, −0.152)	<0.001	
	Total	−0.316 (−0.407, −0.230)	<0.001	
Profile 3 → Resilience → Environment quality of life	Indirect	−0.091 (−0.141, −0.052)	<0.001	24.2%
	Direct	−0.285 (−0.393, −0.174)	<0.001	
	Total	−0.376 (−0.482, −0.270)	<0.001	

*Note*: Standardized coefficients for the direct and indirect effects of the latent profiles compared with Profile 1 on the outcome variables of problematic alcohol use and quality of life are presented. Direct effects represent the effects of Profiles 2 and 3 on each outcome variable that were not explained by resilience. Indirect effects represent the effects of Profiles 2 and 3 on each outcome variable that were explained by resilience. The proportion mediated represents the percentage of the total effect of a predictor variable on an outcome mediated by resilience. Profile 1 represents Low Impulsivity/Urgency. Profile 2 represents Medium Impulsivity. Profile 3 represents High Impulsivity/Urgency. The study covariates, which included age, sex, race, marital status, years of education, and annual household income, were entered into the multivariable model in a single step.

### Supplemental analysis

To demonstrate the advantage of the LPA approach over the traditional variable‐centered approach, we conducted a supplemental analysis examining the associations between impulsivity facets and problematic alcohol use using a multivariable linear regression model with all eight impulsivity facets simultaneously included in the model. Estimates from this model would reveal the unique effect of each impulsivity facet on problematic alcohol use, while controlling for the effects of the other impulsivity facets. As shown in Table 6, only three impulsivity facets (BIS attentional, BIS nonplanning, and UPPS‐P negative urgency) were uniquely associated with problematic alcohol use in the expected positive direction, whereas the other impulsivity facets, including positive urgency, were not associated with problematic alcohol use. These findings contrasted with the LPA results, which showed that most of the impulsivity facets (except sensation seeking) were well‐differentiated between latent profiles and that elevations in both negative and positive urgency scores were key characteristics of Profile 3. This supplemental example illustrated the novelty of the LPA approach as a useful method to model within‐person clustering of impulsivity facets and associations with problematic alcohol use.

**TABLE 6:** Supplemental analysis using a traditional variable‐centered multivariable regression.

	*b*	95% Confidence intervals	*p*	VIF
Barratt Impulsiveness Scale				
Attentional	0.34	0.04, 0.63	0.027	2.48
Motor	−0.07	−0.36, 0.21	0.622	2.04
Non‐planning	0.63	0.38, 0.88	<0.001	2.70
UPPS‐P Impulsive Behavior Scale				
Negative urgency	8.50	6.27, 10.73	<0.001	3.81
Lack of premeditation	−0.62	−3.14, 1.89	0.626	2.12
Lack of perseverance	−3.47	−5.95, −0.98	0.006	2.26
Sensation seeking	−0.34	−1.83, 1.14	0.652	1.21
Positive urgency	0.01	−1.90, 1.92	0.990	2.68

*Note*: Only three impulsivity facets (BIS attentional, BIS nonplanning, and UPPS‐P negative urgency) were significantly associated with problematic alcohol use in the expected positive direction. Although the Variance Inflation Factor (VIF, a measure of multicollinearity) values were generally within the acceptable range, the significant inverse association between lack of perseverance and problematic alcohol use was unexpected and difficult to interpret. This is particularly the case given that as a single predictor of problematic alcohol use, lack of perseverance was significantly and positively associated with the outcome (*b* = 9.13, 95% confidence intervals = 6.95, 11.31, *p* < 0.001). This supplemental analysis illustrated some potential problems with the application of a traditional variable‐centered multivariable regression in modeling eight impulsivity facets together, and bolstered the rationale for using LPA as an alternative analytic approach to model within‐person clustering of impulsivity facets.

## DISCUSSION

Leveraging the availability of two widely used trait impulsivity measures in the same dataset (BIS and UPPS‐P), we identified three impulsivity profiles (Low Impulsivity/Urgency, Medium Impulsivity, and High Impulsivity/Urgency) based on eight indicators of impulsivity through a data‐driven analytic approach. The LPA findings generally aligned with prior research that highlighted the unique role of urgency facets in affective or emotional regulation and substance use risk (Cyders & Smith, 2008; King et al., 2018; Smith & Cyders, 2016). However, our study extended these findings by revealing how facets of impulsivity clustered within individuals. That is, individuals who reported high negative urgency were also likely to score high on positive urgency and most of the other impulsivity facets, an insight that was found only through the LPA approach. The current findings showed that the Medium Impulsivity and High Impulsivity/Urgency Profiles had markedly higher rates of past‐year AUD than the Low Impulsivity/Urgency Profile (87.4% and 100% versus 37.7%). Analysis of the AUDIT score using clinical cutoffs showed a consistent pattern, in which 85.2% of those with Medium Impulsivity had AUDIT ≥8 and 100% of those with High Impulsivity/Urgency had AUDIT ≥16. These findings aligned with prior research showing a positive association between impulsivity and AUDIT score in AUD (Szczypiński et al., 2021) and nonclinical samples (Maccombs‐Hunter & Bhat, 2022), and highlighted the critical role that clustering of multiple impulsivity facets (particularly the urgency facets) had on problematic alcohol use.

The allostasis model of addiction postulates that as AUD progresses, neuroadaptations due to excessive alcohol use would promote a shift from reward‐driven drinking in the positive reinforcement phase to relief‐driven drinking in the negative reinforcement phase (Koob & Le Moal, 2005). Elevations in negative urgency in the High Impulsivity/Urgency Profile provided evidence that converged with the allostasis model wherein individuals may impulsively engage in drinking behaviors in the face of negative emotions (Vendruscolo et al., 2023; Zorrilla & Koob, 2019). Indeed, clinical research has documented that individuals who drink primarily for relief rather than reward or habit reported greater alcohol use and craving, heavier drinking, and more negative consequences over a 12‐week period (Grodin et al., 2024). Expanding beyond negative urgency, a novel finding of the LPA is the concurrent elevation of positive urgency in the High Impulsivity/Urgency Profile. Consistent with recent research indicating that drinking motivated by reward and pleasurable effects of alcohol remained prominent among individuals with AUD (King et al., 2025), the elevation of positive urgency in the High Impulsivity/Urgency Profile highlighted impulsive drinking behavior in the face of positive emotions as a relevant factor in the maintenance of problematic alcohol use (Heilig, 2025).

Of all the indicators included in the LPA, sensation seeking, referring to the tendency to pursue novel and stimulating experiences (Hittner & Swickert, 2006), showed the least variability across latent profiles and was therefore less useful in inferring risk for problematic alcohol use in the current sample of adult participants. Prior research showed that sensation seeking was the only UPPS‐P facet not correlated with some of the BIS subscales (Wardell et al., 2016). Moreover, sensation seeking may follow a distinct developmental trajectory in which it peaks during late adolescence and young adulthood and then declines with age (Argyriou et al., 2020; Steinberg et al., 2008). One potential explanation for the less variable nature of sensation seeking in the present adult sample (with a mean age of 42.5 years) lies in how this facet of impulsivity was assessed. For example, items used to measure sensation seeking included “I would enjoy water skiing” and “I would enjoy parachute jumping” which may have different incentives for different age groups depending on physical health and personal responsibilities (Liu et al., 2020).

The inverse association between impulsivity and resilience is consistent with prior work suggesting that individuals high in urgency may face challenges in regulating their responses during periods of intense emotion (Cyders & Smith, 2008), reducing their capacity to adaptively cope with adversity. Our mediation analysis provided additional insight by showing resilience as a pathway leading to problematic alcohol use and low quality of life. It is important to note that the proportion mediated was higher for quality of life outcomes (18.1%–27.3%) than for problematic alcohol use (8.7%–9.1%), suggesting that strength‐based interventions designed to enhance resilience may be particularly suited for promoting quality of life outcomes within the context of alcohol intervention efforts. The benefits of resilience interventions on stress, anxiety, depression, and quality of life (Blessin et al., 2022) are especially relevant given the advantages in accessibility of many strength‐based interventions, which can be done online in brief, self‐led formats and can benefit a variety of populations (Bolier et al., 2013; Sin & Lyubomirsky, 2009), potentially decreasing the stigma associated with these interventions compared with typical AUD treatment. A recent meta‐analysis of positive psychology interventions implemented to aid substance use recovery showed feasibility and initial promise, though the effect sizes were small and nonsignificant (Carlon et al., 2025). To advance this line of work, future research should assess whether strength‐based interventions that aim to enhance quality of life (e.g., ABC PLEASE Skill in dialectical behavior therapy [DBT]) can be integrated within a broader addictions intervention framework to facilitate behavior change and support recovery from AUD (Luk & Thompson, 2024).

This study has several strengths, including the LPA approach, a noncollege student sample with a large group of individuals with AUD, and the inclusion of multidimensional quality of life outcomes, an integral part of recovery from AUD (Hagman et al., 2022). While the LPA approach yielded a three‐profile solution that may be viewed as equivalent to trichotomizing the data, it is a data‐driven analytic approach that avoids the use of arbitrary cutoffs. In our view, the LPA approach, especially when applied to eight impulsivity facets, represents a meaningful empirical extension of prior LPA studies of the UPPS‐P, and the translational value of the current findings to inform clinical practice rests on its remarkably strong associations with problematic alcohol use and past‐year AUD. Despite these strengths, several limitations should be acknowledged. First, a major limitation of this study is that, due to the cross‐sectional study design, the timing of variables cannot be determined, which limited inferences that can be drawn in the mediation model regarding the direction of effects. While the pathways implied in the mediation model were conceptualized based on theory (i.e., that impulsivity traits tend to be stable characteristics and resilience can be targeted and changed), the associations among impulsivity, resilience, and alcohol‐related outcomes may be bi‐directional and dynamic, warranting future longitudinal research to replicate and extend the current findings. Second, guided by our research questions, which mapped onto the gap in existing literature, our analysis focused on a single mediational pathway through resilience and did not account for other potential pathways, such as those originating from family history of AUD and childhood trauma or through internalizing symptoms. Future research can build upon our findings to test a mediation model with multiple positive and negative pathways to alcohol‐related outcomes. Third, self‐reported measures may be subject to recall bias, and the use of multiple assessment methods can be considered for future research. Fourth, our analyses also did not control for time in recovery or time since last alcohol use. Fifth, we utilized trait measures of impulsivity and resilience in this study, which did not capture potential moment‐to‐moment fluctuations in these variables. Future research can address this limitation by measuring state‐level variations in facets of impulsivity and resilience using novel sampling methods such as ecological momentary assessment to improve prediction of risk for alcohol misuse and impaired quality of life in real‐world settings.

## CONCLUSION

Using LPA, we identified three groups of individuals with varying levels of trait impulsivity facets, with the Medium Impulsivity and High Impulsivity/Urgency Profiles exhibiting lower resilience, higher problematic alcohol use, and lower quality of life. As the Medium Impulsivity and High Impulsivity/Urgency Profiles were most differentiated from the Low Impulsivity Profile on both negative and positive urgency, our findings highlighted the need to target emotion‐based impulsivity and increase emotional regulation skills to address problematic alcohol use and well‐being outcomes (e.g., STOP and TIP Skills in DBT; Luk & Thompson, 2024). Furthermore, resilience was identified as a mediator of the associations between latent profiles and alcohol‐related outcomes, highlighting resilience as a potential intervention target in addiction treatment. Mindfulness‐based and positive psychology interventions aimed at enhancing resilience may be beneficial for individuals with moderate to high impulsivity to manage their drinking behavior and improve their quality of life. More clinical research is needed to test the optimal way to integrate resilience training into existing evidence‐based interventions for addictive behaviors (e.g., cognitive behavioral therapy and motivational enhancement therapy) and inform personalized alcohol treatment.

## FUNDING INFORMATION

This study was supported by the Intramural Research Program of the National Institutes of Health (NIH), the National Institute on Alcohol Abuse and Alcoholism Division of Intramural Clinical and Biological Research (Z1A AA000130), and by the National Institute of Mental Health supporting Matthew Thompson (T32 MH078788). The contributions of the NIH authors were made as part of their official duties as NIH federal employees, are in compliance with agency policy requirements, and are considered Works of the United States Government. However, the findings and conclusions presented in this paper are those of the authors and do not necessarily reflect the views of the NIH or the US Department of Health and Human Services.

## CONFLICT OF INTEREST STATEMENT

The authors do not have any conflicts of interest to disclose.

## Data Availability

The data presented in this article are not readily available because of ethical concerns regarding patient privacy and original patient consent.
